# Texas’ Community Health Workforce: From State Health Promotion Policy to Community-level Practice

**Published:** 2005-10-15

**Authors:** Donna C Nichols, Cecilia Berrios, Haroon Samar

**Affiliations:** Center for Policy and Innovation, Texas Department of State Health Services; Ms. Nichols was previously Director of Health Promotion at the Texas Department of Health and the staff director for ensuring implementation of the lay health education workforce statute in Texas; Texas Department of State Health Services, Austin, Tex; Texas Department of State Health Services, Austin, Tex

## Abstract

**Background:**

Imagine yourself in Texas as a newly arrived immigrant who does not speak English. What would you do if your child became ill? How would you find a doctor? When you find one, will the doctor speak your native language or understand your culture? In a state of approximately 22 million people, many Texas residents, marginalized by poverty and cultural traditions, find themselves in this situation. To help them, some communities across Texas offer the services of *promotores*, or community health workers, who provide health education and assist with navigating the health care system.

**Context:**

In 1999, Texas became the first state in the nation to recognize these workers and their contributions to keeping Texans healthy. This paper examines a state health promotion policy that culminated in a training and certification program for *promotores* and the impact of this program on the lay health education workforce in Texas.

**Methods:**

In 1999, the Texas legislature established the 15-member *Promotor(a)* Program Development Committee to study issues involved in developing a statewide training and certification program. During its 2-year term, the committee met all six of its objectives toward establishing and maintaining a *promotor(a)* certification program.

**Consequences:**

By the end of December 2005, it is estimated that there will be more than 700 certified *promotores* in Texas. State certification brings community health workers into the public health mainstream as never before.

**Interpretation:**

*Promotores*, a community health safety net and a natural extension of the health and human services agencies, improve health at the neighborhood level. Certification brings renewed commitment to serving others and a distinction to those who have been the unsung heroes of public health for decades.

## Background

There are about 30 different names for lay individuals who provide community-level health services in the United States (1). In Texas, these lay health educators are called *promotores* or *community health workers*. The term *promotores* refers to lay health educators who provide services in particular along the Texas–Mexico border; the term *community health worker* (*CHW*) refers to lay health educators who practice anywhere in Texas. *Promotores* or CHWs are often vital in linking underserved and disenfranchised clients with essential health and human services. The uniqueness of their service lies in their ability to relate to clients through shared experiences drawn from living in the same communities. Firsthand knowledge of the barriers that affect the health of a community gives CHWs a stake in eliminating those barriers (2-5). Officially, Texas law defines a *promotor(a)* or CHW as a person who, with or without compensation, provides cultural mediation between communities and health and human services systems, informal counseling and social support, and culturally and linguistically appropriate health education; advocates for individual and community health needs; ensures that people get the health services they need; builds individual and community capacity; or provides referral and follow-up services (1).

The role of a *promotor(a)* or CHW differs widely from community to community depending on the needs each community identifies. For example, CHWs may serve as interpreters for clients during physician visits, help clients identify benefits for which they are eligible, and assist them to complete applications to receive benefits and services. As community leaders, they may empower their neighbors by organizing and motivating them to become actively involved in improving living conditions within their neighborhood. In the role of health educators, CHWs may inform their clients of ways to prevent illness and disease and teach them how to manage chronic diseases. Experience has shown that CHWs are a valuable resource for informing their neighbors and recruiting them to participate in social programs for which they qualify.

In 1999, Texas became the first state in the nation to legislate a statewide voluntary *promotor(a)*or CHW training and certification program. As part of that legislation, the state established a committee under the direction of the Texas Department of Health (TDH) to study the feasibility and elements of such a program and make recommendations for its implementation. This paper describes the work of this committee and the resulting certification program for *promotores* and CHWs in Texas.

## Context

The process of creating a statewide, state-supported training and certification program for *promotores* and CHWs involved three groups. First, in the mid-1990s, a series of meetings was held that brought together *promotores* or CHWs, a CHW alliance, community leaders, health professionals, and others interested in this public health work force from several southwestern border states. Second and also important were the *promotor(a)* organizations, formed to provide communication and sharing networks among *promotora* programs. One example is the South Texas *Promotora* Association, a loose federation of *promotores* from 11 programs in the Lower Rio Grande Valley. One role of these organizations was to advocate locally, regionally, and statewide for recognition of their work. Third and finally, a group of state legislators, all representing districts that form the border with Mexico, became catalysts for creating a formal means through which to recognize and legitimize *promotores'* work. All three of these groups helped to provide the context for the initial legislation (6).

## Methods

House Bill 1864, enacted by the 76th Texas Legislature in May 1999, directed the TDH to establish a temporary committee to make recommendations on issues involved in the voluntary training and certification of *promotores* or CHWs (4). From this directive, the *Promotor(a)* Program Development Committee (PPDC) was formed. The PPDC was composed of 15 members, as provided for by the legislation: two *promotores*, two members of the general public, two employees of the TDH, seven representatives of designated colleges and universities in Texas, one representative of the Texas Workforce Commission, and one representative of the Texas–Mexico Border Health Services Delivery Project (7). The committee was charged with the following six tasks:  

- Review and assess *promotor(a)* or CHW programs currently in operation around the state;
- Study the feasibility of establishing a standardized curriculum for *promotores* or CHWs;
- Study the options for certification of *promotores* or CHWs and the settings in which certification may be appropriate;
- Assess available methods to evaluate the success of *promotor(a)* or CHW programs;
- Create, oversee, and advise local pilot projects established under this article, subject to the availability of appropriations;
- Evaluate the feasibility of seeking a federal waiver so that *promotor(a)* or CHW services may be included as a reimbursable service provided under the state Medicaid program (7).

The PPDC met monthly for 2 years and submitted a report on its activities after 1 year. The committee's work was completed in 2001.

## Consequences

### Program review and assessment

The first charge, to review and assess *promotor(a)* or CHW programs currently in operation around Texas, required the PPDC members to identify programs that train and employ *promotores* or CHWs within their organizational networks. Using a *Promotor(a)* or CHW Workforce and Training Questionnaire, the PPDC found approximately 30 existing programs using some 300 *promotores* or CHWs as paid or unpaid, full-time or part-time staff. *Promotores* or CHWs were serving in neighborhood clinics, local health departments, community-based organizations, faith-based agencies, and university-sponsored activities.

### Curriculum development

The second charge asked PPDC members to study the feasibility of establishing a standardized curriculum for *promotores* and CHWs. PPDC members found that each existing training program uses its own curriculum, which tends to focus on health specialties, organizational standards, and other issues shaped by the needs of the community the program serves. These curricula are as diverse in content and number of course hours as the programs themselves. Consequently, a *promotor(a)* or CHW may be well trained to work with the agency where training occurred but lack the skills required by a different agency. Moreover, the differences in training can lead to uncertainty as to what basic competencies potential employers can expect.

The PPDC reviewed state and national curricula and decided that implementation of standard curriculum guidelines, which instill portable skills, would ensure a common base of knowledge and guarantee certain basic skills. This standardized curriculum focused on eight core areas of competence (1):

- Advocacy
- Interpersonal relations
- Capacity building
- Communication
- Knowledge
- Organization
- Teaching
- Service coordination

These competencies are critical to accomplishing community health improvement goals, including chronic disease prevention.

Minimum standard learner-centered objectives were created for each competency. Stakeholder feedback was sought to make sure these objectives were realistic and representative of *promotor(a)* or CHW daily activities. With this the state is better able to ensure uniformity and transferability of basic knowledge and skills regardless of where the *promotor(a)* or CHW practices (8).

Public hearings were held in the cities of Arlington, El Paso, Houston, and Weslaco on proposed rules for the certification of *promotores* or CHWs. More than 150 individuals commented, with the majority being *promotoras* or CHWs who participated in the public hearings. A diverse group of organizational and political stakeholders were also represented, including representatives from colleges and universities, government agencies, and community health coalitions (2). The rules for certification adopted by the Texas Board of Health in July 2000 are the result of the combined input of the TDH, the PPDC, and the many community members who participated in the process. The rules serve as a blueprint for the training and certification program.

Among other qualifications, a minimum of 160 course hours must be offered for a curriculum to qualify for certification. To be grandfathered into certification, individuals must submit an application and must have performed *promotor(a)* or CHW services not fewer than 1000 cumulative hours from July 1997 to January 2004. This certification process was based loosely on the professional certification process for health educators known as the Certified Health Education Specialist and administered by The National Commission for Health Education Credentialing, Inc.

### Options and settings for certification

The third charge required the PPDC to study the options for certification of *promotores* or CHWs and the settings in which certification may be appropriate. The committee, with stakeholder feedback, chose to certify *promotores* or CHWs, their instructors, and sponsoring institutions or training programs. The rules specify qualifications and special provisions for those who have historically been a part of the CHW movement. Settings that are safe and comfortable and where learners feel valued and respected were given primary consideration to support the special needs of these adult learners.

### Program evaluation methods

The fourth charge to the PPDC was to assess available methods to evaluate the success of *promotor(a)* or CHW programs. Findings indicated that programs use evaluation tools differently. Some programs evaluate processes and others focus on outcomes. Some programs have used a combination of methods, and some programs do not gather data at all or use data to evaluate their efforts. The PPDC agreed that as an overriding principle, programs should at least be able to integrate an evaluation component that is adaptable for the varied *promotor(a)* or CHW functions, including health, social services, education, or instruction. Likewise, the ongoing evaluation of the program for practical purposes should include the ability to assess curriculum, certification, training, and programmatic implementation. In response to theses findings, the PPDC initially recommended the use of a comprehensive, thoroughly field-tested evaluation package known as the *Community Health Worker Evaluation Tool Kit*, developed by the University of Arizona Rural Health Office and the College of Public Health. The TDH (renamed The Texas Department of State Health Services in September 2004) is in the initial phase of designing a tool to evaluate program processes and outcomes.

### Pilot projects

The fifth charge to the PPDC was to create, oversee, and advise local pilot projects established under this article, subject to the availability of appropriations that may be used for this purpose. Five pilot sites were selected by the TDH and Health and Human Services Commission (HHSC) committee through a competitive process. However, because of shortfalls in general revenue, the pilot sites were unfunded, which precluded the development of neighborhood projects to test the feasibility of training, certifying, and employing CHWs. 

However, in late 2002, HHSC assisted the TDH with funding a neighborhood project by seeking foundation resources to obtain additional federal Children's Health Insurance Program (CHIP) matching funds. In November 2002, Rockwell Fund, Inc awarded $25,000 to Harris County Hospital District, Gateway to Care in Houston, and this contract was executed in June 2003. The purpose of the pilot is to test the effectiveness of CHWs in increasing access to primary and preventive health care and reducing overall health care costs to the state. This project received an additional award of $173,000 from the Houston Endowment for a 3-year period.

During the first 14 months of implementation of the Harris County pilot project, 1017 CHIP and Medicaid families were served by receiving information on and assistance with using health resources. Eight *promotores* or CHWs and one instructor were certified by the TDH to work with Gateway to Care families. In addition, Gateway to Care was approved by the TDH as a certified training program.

### Reimbursement of services from Medicaid

The sixth charge to the PPDC was to evaluate the feasibility of seeking a federal waiver so that *promotor(a)* or CHW services would be included as reimbursable services provided under the state Medicaid program. The PPDC recommended that all practical sources of funding within the state be considered in supporting CHW services. 

The PPDC recommended the following changes within the Medicaid system and "right steps" for appropriate health and human services commission agencies to take:

- Apply best practice models to eliminate barriers to care. These included employing or empowering *promotores* or CHWs to assist recipients in accessing Medicaid services, simplifying Medicaid eligibility policies and procedures, reducing documentation required by the application process, and requiring customer service and cultural competency standards;
- Enable community residents to collaborate with health and human services systems to build or tailor the Medicaid infrastructure to the unique conditions of their environment;
- Promote independence and local control among community residents and sustain commitment among health and human services agencies to improve quality of life and eliminate health disparities.

### Certification of *promotores* or CHWs in Texas

**Recent legislation**

The PPDC accomplished its objectives in 2001 of preparing for a *promotor(a)* training and certification program. In that same year, the Texas legislature passed two pieces of legislation related to *promotor(a)* certification. Senate Bill 1051 mandated that all *promotores* or CHWs who receive compensation for their services be certified. Previously, the certification process was voluntary for all *promotores* or CHWs. The second piece of legislation, Senate Bill 751, required that state health and human services agencies use certified *promotores* to the extent possible for recipients of medical assistance. Together, these mandates increased the immediate need for approved training programs and a standardized certification process.

***Promotor(a) *or CHW Training and Certification Advisory Committee**

To oversee the certification process, the Texas *Promotor(a)* or CHW Training and Certification Advisory Committee was established in 2001. This committee, reporting to the TDH, determines the eligibility of and recommends certification for *promotores* or CHWs, instructors, and sponsoring institutions or training programs.

The *Promotor(a)* or CHW Training and Certification Advisory Committee is composed of nine members approved by the Texas Health and Human Services Commission. (Prior to September 1, 2004, members were approved by the former Texas Board of Health.) The committee includes four certified *promotores, *CHWs, or the equivalent; two members of the public; one member from the Texas Higher Education Coordinating Board or a higher education faculty member who has teaching experience in community health, public health, or adult education and has trained *promotores* or CHWs; and two professionals who work with *promotores* or CHWs in a community setting (2).

By December 2002, the committee had developed, field tested, and finalized the certification application form for *promotores* or CHWs. Six certifications were conferred at an official ceremony at the 2002 CHW state conference, and the committee conducted several promotional workshops to distribute certification applications and instructions (available from www.tdh.state.tx.us/ophp/chw/chwdocs.htm). By December 2003, a database for tracking the review and disposition of applications for all three forms of certification and recertification was implemented; 224 certifications for *promotores* or CHWs were conferred; certification IDs were accepted as proof of qualifications by all organizations in Texas; certification renewal forms were created; and a Web site for the Texas *Promotor(a)* or CHW Training and Certification Advisory Committee was launched. By December 2004, the committee had certified 337 *promotores* or CHWs, 24 instructors, and 3 training programs. By the end of December 2005, it is estimated that there will be more than 700 certified *promotores* or CHWs in Texas.

## Interpretation

The impact of the training and certification program on CHWs is deeply personal. CHWs provide a number of reasons for seeking certification: self-development, recognition by others of their position and work, professional enhancement, new incentives to work, and the possibility for career development (6). In addition, certification of CHWs provides credibility, recognition, and the development of scope of practice.

Counterarguments are made by CHWs who wish to function on a volunteer basis or by individuals who are concerned that certification of CHWs will erode the professional base of another regulated, licensed, or certified professional group. There are many people who feel that professionalizing CHWs will result in a loss of the indigenous qualities that contribute to their success. However, CHWs may continue to volunteer their services without penalty; CHWs who provide services without compensation are not required to be certified.

CHWs in Texas, for the most part, work in an integrated fashion within the health and human services system and seldom work with a specific "carve-out" or solely funded CHW program. Therefore, sustainability of CHW programs may not be a major issue for Texas. As with all federally funded or state or locally funded programs, sustainability is an issue regardless of the types of individuals providing services to their communities. Institutions are at a greater legal risk if their CHWs are not certified, because many of these workers visit clients in their homes and are at greater personal risk if they cannot visibly and legitimately identify themselves with an organization. CHWs in Texas are just beginning to receive compensation for their services through various sources. For example, certified CHWs have been employed by Maximus, Texas' Medicaid enrollment broker, to conduct the outreach necessary to inform Medicaid enrollees of their benefits.

There is no application fee associated with certification. Costs, if any, are borne by the employer or the CHW for training or recertification, which may include a cost for continuing education. To date, cost has not been an issue. The greater issue in the future is anticipated to be access to and availability of continuing education for CHWs. The time needed for training has not proven to be a barrier to certification, either. CHWs who wish to improve their skills and knowledge, regardless of certification status, will have the opportunity to do so through certified training programs.

The greatest challenge to implementing the CHW training and certification program was working with a diverse, vocal, and broad-based committee that represented academic systems, state agencies, the general public, and CHWs in creating a shared vision and a unified set of recommendations on how this training and certification program should function. Staff worked diligently to ensure that the voice of the CHW was heard throughout the process and established a public comment period as a standard procedure for each official meeting and hearing conducted by the TDH.

Since the rollout of the certification application for *promotores* or CHWs in December 2002, there has been much interest in the certification program statewide. Program staff respond to approximately 120 inquiries per month about certification policies and procedures. As of May 2005, the Texas Department of State Health Services had certified 500 *promotores*, 24 instructors, and 6 sponsoring institutions or training programs in Texas. Two additional training programs were certified in June 2005 (Table). A map showing the number of certified *promotores* in each Texas county is available from www.tdh.state.tx.us/ophp/chw/pubs/promotorasmay05.pdf*.

For the first time, Texas has recognized the power and the value of this community health safety net by giving long overdue recognition to the health education workforce that has worked silently and tirelessly to keep their communities healthy and fit.

## Figures and Tables

**Table:** Certified *Promotor(a)* or Community Health Worker (CHW) Training Programs, Texas, 2004

**Training Program**	**City**
El Paso Community College	El Paso
South Texas College	McAllen
Gateway to Care	Houston
Houston Community College	Houston
The Rose Imaging Center/The Empower Her Project	Houston
City of Fort Worth Public Health Department	Fort Worth
